# Analyzing Main and Interaction Effects of Length of Stay Determinants in Emergency Departments

**DOI:** 10.15171/ijhpm.2019.107

**Published:** 2019-11-16

**Authors:** Gorkem Sarıyer, Mustafa Gökalp Ataman, İlker Kızıloğlu

**Affiliations:** ^1^Department of Business Administration, Yaşar University, İzmir, Turkey.; ^2^Department of Emergency Medicine, Çiğli Regional Training Hospital, İzmir, Turkey.; ^3^Department of General Surgery, Çiğli Regional Training Hospital, İzmir, Turkey.

**Keywords:** Emergency Department, Length of Stay, Mode of Arrival, Clinical Acuity, Factorial ANOVA

## Abstract

**Background:** Measuring and understanding main determinants of length of stay (LOS) in emergency departments (EDs) is critical from an operations perspective, since LOS is one of the main performance indicators of ED operations. Therefore, this study analyzes both the main and interaction effects of four widely-used independent determinants of ED-LOS.

**Methods:** The analysis was conducted using secondary data from an ED of a large urban hospital in Izmir, Turkey. Between-subject factorial analysis of variance (ANOVA) was used to test the main and interaction effects of the corresponding factors. *P* values <.05 were considered statistically significant.

**Results:** While the main effect of gender was insignificant, age, mode of arrival, and clinical acuity had significant effects, whereby ED-LOS was significantly higher for the elderly, those arriving by ambulance, and clinically-categorized high-acuity patients. Additionally, there was an interaction between the age and clinical acuity in that, while ED-LOS increased with age for high acuity patients, the opposite trend occurred for low acuity patients. When ED-LOS was modeled using gender, age, and mode of arrival, there was a significant interaction between age and mode of arrival. However, this interaction was not significant when the model included age, mode of arrival, and clinical acuity.

**Conclusion:** Significant interactions exist between commonly used ED-LOS determinants. Therefore, interaction effects should be considered in analyzing and modelling ED-LOS.

## Background

Emergency departments (EDs) are the most critical medical resource for delivering emergency services. Given the busy work environments within EDs, and the need to make immediate decisions to ensure prompt treatment of patients, medical errors or inadequate service are unavoidable.^1^ Timeliness is thus a major quality-of-care indicator for ED services, on par with patient centeredness, guideline concordance, efficiency and equity, number of return visits within 30 days, etc.^2^ Timeliness of ED services is mainly measured by length of stay in EDs (ED-LOS).^3,4^ Given that ED-LOS is a challenge for ED management, understanding its main determinants is a key issue for dealing with this problem. Consequently, analyzing and reporting these factors have attracted significant attention recently.

Table 1 presents a summary of previous studies analyzing the effects of different factors on ED-LOS.

**Table 1:** Previous Studies of ED-LOS Determinants

**Factor Levels**	**Independent Factors**	**Literature Studies**	
		**Significant Factor**	**Non-significant Factor**
Demographic/patient	Gender	^ 5-7 ^	^ 8-14 ^
	Age	^ 5, 9, 11-13, 15-21 ^	^ 8, 10, 14, 22 ^
	Race	^ 8, 10, 14, 23, 24 ^	^ 13 ^
	Type of health insurance	^ 8, 13, 25 ^	^ 14 ^
	Language	^ 26 ^	^ 27 ^
Clinical	Clinical acuity or triage category	^ 5, 7-9, 11, 12, 18, 21, 22, 28-30 ^	^ 31 ^
	Mode of arrival	^ 11, 12, 17, 19, 21, 22 ^	
	Use of diagnostic test or laboratory studies	^ 6, 10, 28, 32-36 ^	
ED	ED census	^ 6, 9, 37-39 ^	^ 8, 12 ^
	Number of physicians	^ 8, 32 ^	
	Number of support personnel	^ 8 ^	
	Training level of ED personnel	^ 8 ^	^ 10 ^
Time	Day of visit	^ 12, 18 ^	^ 6, 9, 23, 25 ^
	Month of visit		^ 9, 12 ^
	Period of visit	^ 12, 17, 18, 40 ^	^ 6, 23, 36 ^
System/organizational	Geographic location	^ 10, 29 ^	^ 14 ^
	Hospital ownership	^ 23 ^	^ 10 ^

Abbreviations: ED, emergency department; ED-LOS, length of stay in emergency department.

Previous studies have analyzed the main effects on ED-LOS of many different patient, physician, time, or organizational factors. However, besides the main or individual effects of each factor, 2 or more factors may interact with significant effects on ED-LOS. Interaction effects represent the combined effects of factors on the dependent factor. Specifically, the impact of one independent variable depends on the level of the other independent variable. To illustrate this in an ED setting, consider a situation where the dependent factor is ED-LOS, and the 2 independent factors are gender and age. Assume ED-LOS is measured in minutes on a continuous scale while the independent factors are measured on nominal scales with 2 levels each: male and female for gender, and young and old for age. Assume further that the main effect of age on ED-LOS is significant whereas gender has no significant effect. That is, ED-LOS differs significantly between young and old patients but not between males and females. However, if the effect of age on ED-LOS for males is different from the effect of age on ED-LOS for females then there is an interaction, which needs to be considered while making plans for ED operations. This is because the ED-LOS of old male and female patients is likely to differ and it is also likely to differ for young male and female patients.

This study therefore analyzed both the main and interaction effects of 4 widely-used independent determinants of ED-LOS (gender, age, mode of arrival, and clinical acuity) using between-subject factorial analysis of variance (ANOVA). Analyzing these interaction effects can offer deeper insights for ED managers and practitioners while planning operations than just focusing on main effects.

## Methods

The analysis was conducted using secondary data for all patient arrivals during May 2017 at the ED of a large urban hospital, with an annual rate of 350 000 visits approximately, in Izmir, Turkey. The sample size was adequate to investigate the study’s objectives.

The independent variables were gender, age, mode of arrival, and clinical acuity while the dependent variable was ED-LOS, defined as the time from registration to exit (ie, the patient is admitted to a hospital bed, transferred to another department or a different hospital, or goes home).^3,4^ These variables were chosen because they are the most commonly used in ED-LOS research. While ED-LOS was a continuous variable measured in minutes, the independent variables were all categorical in accordance with methodological assumptions. The independent variables had the following levels:

- Gender: male, female
- Age: age ≤14, age: [15-64], age ≥65
- Mode of arrival: walk-in, by ambulance
- Clinical acuity: high, low

Clinical acuity was based on the triage categories assigned to patients by the responsible nurses in the triage room. The studied ED uses a 3-level scale from 1 to 3 of emergent, urgent, and non-urgent patients.^41^ Patients with life or limb-threatening problems that require immediate intervention are classified as emergent; patients requiring prompt care but not at risk of loss of life or limb if left untreated for several hours are classified as urgent; patients who require treatment but are not time-critical are classified as non-urgent. In this study, since time is a critical factor for emergent and urgent patients, they were labeled as high acuity while those triaged as not urgent were labeled as low acuity.

The analysis included 4 main steps:

*Step 1:* The raw data was pre-processed and structured. During preprocessing, entries with missing values, inconsistent, or redundant data, and outliers were removed. The standardization rule was used for outlier detection. According to this rule, the dependent variable, ED-LOS, was standardized by converting each patient’s ED-LOS values to standard z-scores (following a standard normal distribution with a mean of 0 and a standard deviation of 1). Standardized z-scores falling outside the 99% CI (-2.5, +2.5) were considered outliers.

*Step 2:* The study hypotheses were defined.

*Step 3:* The required assumptions for between-subject factorial ANOVA were checked.

*Step 4:* Based on the number of independent variables and the levels of each, the appropriate between-subject ANOVA designs were defined and tested.

Since 4 main independent factors were considered and their 3-way effects on ED-LOS were to be investigated, 4 hypotheses sets were described. Table 2 shows the set of hypotheses, the independent variables and number of levels, the hypotheses (null versions), and the appropriate ANOVA designs.

**Table 2:** Hypothesis Description

**Set of Hypotheses**	**Independent Variables (No. of Levels)**	**Hypothesis Statements**	**ANOVA Design**
Set 1	Gender (2), age (3), mode of arrival (2)	H_11_: *gender* has a non-significant *main effect* on modelling ED-LOS with the independent variables of set 1.	2*3*2
		H_12_: *age* has a non-significant *main effect* on modelling ED-LOS with the independent variables of set 1.	
		H_13_: *mode of arrival* has a non-significant *main effect* on modelling ED-LOS with the independent variables of set 1.	
		H_14_: *gender* and *age* have a non-significant *interaction effect* on modelling ED-LOS with the independent variables of set 1.	
		H_15_: *gender* and *mode of arrival* have a non-significant *interaction effect* on modelling ED-LOS with the independent variables of set 1.	
		H_16_: *age* and *mode of arrival* have a non-significant *interaction effect* on modelling ED-LOS with the independent variables of set 1.	
		H_17_: *gender, age* and *mode of arrival* have a non-significant *interaction effect* on modelling ED-LOS with the independent variables of set 1.		
Set 2	Gender (2), age (3), clinical acuity (2)	H_21_: *gender* has a non-significant *main effect* on modelling ED-LOS with the independent variables of set 2.	2*3*2
		H_22_: *age* has a non-significant *main effect* on modelling ED-LOS with the independent variables of set 2.	
		H_23_: *clinical acuity* has a non-significant *main effect* on modelling ED-LOS with the independent variables of set 2.	
		H_24_: *gender* and *age* have a non-significant *interaction effect* on modelling ED-LOS with the independent variables of set 2.		
		H_25_: *gender* and *clinical acuity* have a non-significant *interaction effect* on modelling ED-LOS with the independent variables of set 2.	
		H_26_: *age* and *clinical acuity* have a non-significant *interaction effect* on modelling ED-LOS with the independent variables of set 2.	
		H_27_: *gender, age* and *clinical acuity* have a non-significant *interaction effect* on modelling ED-LOS with the independent variables of set 2.	
Set 3	Gender (2), clinical acuity (2), mode of arrival (2)	H_31_: *gender* has a non-significant *main effect* on modelling ED-LOS with the independent variables of set 3.	2*2*2
		H_32_: *clinical acuity* has a non-significant *main effect* on modelling ED-LOS with the independent variables of set 3.	
		H_33_: *mode of arrival* has a non-significant *main effect* on modelling ED-LOS with the independent variables of set 3.	
		H_34_: *gender* and *clinical acuity* have a non-significant *interaction effect* on modelling ED-LOS with the independent variables of set 3.	
		H_35_: *gender* and *mode of arrival* have a non-significant *interaction effect* on modelling ED-LOS with the independent variables of set 3.	
		H_36_: *clinical acuity* and*mode of arrival* have a non-significant *interaction effect* on modelling ED-LOS with the independent variables of set 3.	
		H_37_: *gender, clinical acuity* and*mode of arrival* have a non-significant *interaction effect* on modelling ED-LOS with the independent variables of set 3.	
Set 4	Age (3), clinical acuity (2), mode of arrival (2)	H_41_: *age* has a non-significant *main effect* on modelling ED-LOS with the independent variables of set 4.	3*2*2
		H_42_: *clinical acuity* has a non-significant *main effect* on modelling ED-LOS with the independent variables of set 4.	
		H_43_: *mode of arrival* has a non-significant *main effect* on modelling ED-LOS with the independent variables of set 4.	
		H_44_: *age* and *clinical acuity* have a non-significant *interaction effect* on modelling ED-LOS with the independent variables of set 4.	
		H_45_: *age* and *mode of arrival* have a non-significant *interaction effect* on modelling ED-LOS with the independent variables of set 4.	
		H_46_: *clinical acuity* and *mode of arrival* have a non-significant *interaction effect* on modelling ED-LOS with the independent variables of set 4.	
		H_47_: *age, clinical acuity* and *mode of arrival* have a non-significant *interaction effect* on modelling ED-LOS with the independent variables of set 4.	

Abbreviations: ED-LOS, length of stay in emergency department; ANOVA, analysis of variance.

Statistical analysis was conducted using SPSS version 22.0 for Windows (SPSS, Chicago, IL, USA).

## Results

The total number of patients arriving at the ED during the study period was 30 612. After eliminating missing, inconsistent, and redundant entries, 30 020 entries remained.

Once the outliers had been excluded, based on the ED-LOS values, 29 213 patients’ data remained for the analysis. Thus, 4.57% of the raw data was excluded during pre-processing. Before removing the outliers, the average ED-LOS was 104.22 minutes with a standard deviation of 122.97 minutes. Afterwards, the average ED-LOS was 90.70 minutes with a standard deviation of 87.79 minutes.

### Descriptive Data Analysis

The descriptive statistics of the variables are presented in Table 3.

**Table 3:** Descriptive Statistics

**Independent Variable**	**Levels**	**ED-LOS (min)**		**No. of Patients Per Category (%)**
		**Mean**	**Standard Deviation**	
Gender	Female	93.61	89.03	14 837 (50.79)
	Male	87.68	86.40	14 376 (49.21)
Age	Age ≤14	77.68	75.54	6056 (20.73)
	Age: [15-64]	85.98	84.22	20 647 (70.68)
	Age ≥65	160.88	109.87	2510 (8.59)
Mode of arrival	Walk-in	86.32	84.41	27 884 (95.45)
	By ambulance	182.46	105.42	1329 (4.55)
Clinical acuity	High acuity	124.55	98.65	14 970 (51.24)
	Low acuity	55.11	55.76	14 243 (48.76)

Abbreviation: ED-LOS, length of stay in emergency department.

Table 3 shows that average ED-LOS was longer for females than males and was higher for elderly patients. Similarly, patients arriving by ambulance or with high clinical acuity had noticeably longer ED-LOS values than those who arrived on foot or had low clinical acuity. The standard deviation values indicate that ED-LOS varied significantly within the specific groups. Both females and males, and high and low clinical acuity patients had similar frequencies. However, the frequency distributions differed for age and mode of arrival. Patients in the middle age group [15-64] and those arriving on foot had the highest percentage distributions.

### Hypotheses Testing

A between-subject factorial ANOVA was used to test the main and interaction effects of the independent variables on the dependent variable, ED-LOS. Before applying the method, its required assumptions were tested. Details of the assumptions check are given in Supplementary file 2. The assumptions of normality and homogeneity of variances were both violated (see assumptions 5 and 6). However, since ANOVA works quite well even if the normality assumption is violated (that is, one or more of the distributions are highly skewed) or if the variances differ noticeably between variables,^42^ it was concluded that this method could be used to test the hypotheses.

The study hypotheses were then tested using the proposed designs, based on the number of independent variables and levels of each variable, as shown in the fourth column of Table 2. Table 4 reports the main results of the hypothesis testing while Supplementary file 2 presents the detailed statistics, including sum of squares, degrees of freedom, mean square, F values, *P* values, R-squared and adjusted R-squared values.

**Table 4:** Summarized Results of Hypotheses Testing (*P* Values, Result)

**Sets/Statements**	**Set 1 ( i = 1)**	**Set 2 ( i = 2)**	**Set 3 ( i = 3)**	**Set 4 ( i = 4)**
Statement 1 (H_i1_, i = 1..4)	0.405 (H_11_ is not rejected)	0.116 (H_21_ is not rejected)	0.676 (H_31_ is not rejected)	*P* < .001 (H_41_ is rejected)
Statement 2 (H_i2_, i = 1..4)	*P* < .001 (H_12_ is rejected)	*P* < .001 (H_22_ is rejected)	*P* < .001 (H_32_ is rejected)	*P* < .001 (H_42_ is rejected)
Statement 3 (H_i3_, i = 1..4)	*P* < .001 (H_13_ is rejected)	*P* < .001 (H_23_ is rejected)	0.005 (H_33_ is rejected)	0.019 (H_43_ is rejected)
Statement 4 (H_i4_, i = 1..4)	0.946 (H_14_ is not rejected)	0.063 (H_24_ is not rejected)	0.780 (H_34_ is not rejected)	*P* < .001 (H_44_ is rejected)
Statement 5 (H_i5_, i = 1..4)	0.935 (H_15_ is not rejected)	0.790 (H_25_ is not rejected)	0.972 (H_35_ is not rejected)	0.948 (H_45_ is not rejected)
Statement 6 (H_i6_, i = 1..4)	*P* < .001 (H_16_ is rejected)	*P* < .001 (H_26_ is rejected)	0.101 (H_36_ is not rejected)	0.520 (H_46_ is not rejected)
Statement 7 (H_i7_, i = 1..4)	0.439 (H_17_ is not rejected)	0.499 (H_27_ is not rejected)	0.908 (H_37_ is not rejected)	0.466 (H_47_ is not rejected)

Table 4 shows that the main effects of age (H_12_, H_22,_ H_41_), mode of arrival (H_13_, H_33,_ H_43_), and clinical acuity (H_23_, H_32,_ H_42_) were significant in all models. However, the main effect of gender (H_11_, H_21,_ H_31_) was not significant in any model. Similarly, while the interaction between the age and clinical acuity (H_26,_ H_44_) was significant in both models, the interactions between gender and age (H_14_, H_24_), gender and mode of arrival (H_15_, H_35_), gender and clinical acuity (H_25_, H_34_), mode of arrival and clinical acuity (H_36_, H_46_) were not significant in any model. While the interaction between age and mode of arrival was significant (H_16_) in one model (model between gender, age, mode of arrival), it was not significant (H_45_) in the other model (model between age, mode of arrival, clinical acuity). Finally, none of the 3-way interactions were significant in any model (H_17_, H_27,_ H_37,_ H_47_).

**Figure 1:**
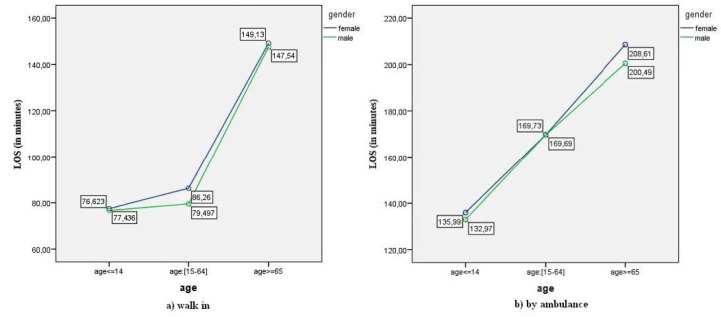
Interaction Plot of Gender, Age, and Mode of Arrival.

**Figure 2:**
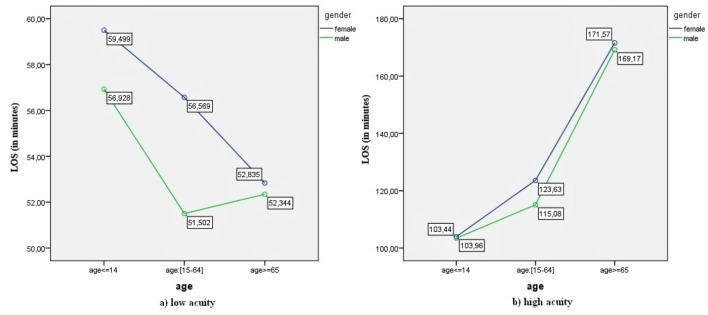
Interaction Plot of Gender, Age, and Clinical Acuity.

**Figure 3:**
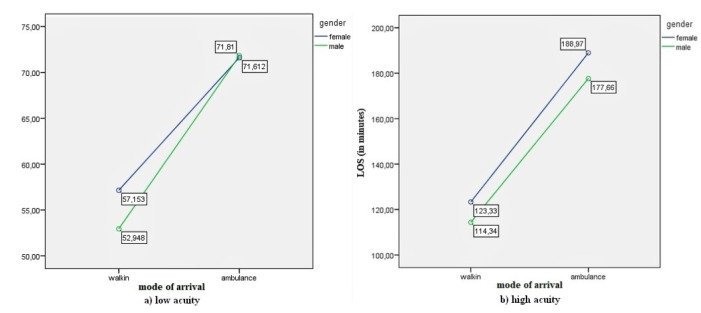
Interaction Plot of Gender, Mode of Arrival, and Clinical Acuity.

**Figure 4:**
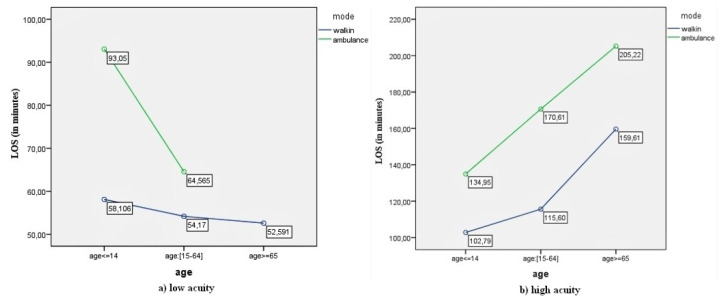
Interaction Plot of Age, Mode of Arrival, and Clinical Acuity.

The interaction plots are presented in Figures 1, 2, 3, and 4.

Figure 1 shows that average ED-LOS was slightly higher for females than males, both for walk-in patients (Figure 1a), and those arriving by ambulance (Figure 1b). ED-LOS increased with age for both genders, regardless of mode of arrival.

Figure 2 shows that ED-LOS differed for the 2 levels of clinical acuity. For low acuity patients, ED-LOS decreased with age for both genders. Although it was highest for the youngest patients (aged ≤14) for both genders, while it was lowest for the middle aged group (age: [15-64]) for males, it was lowest for elderly (aged ≥65) for females (see Figure 2a). On the other hand, for high acuity patients, ED-LOS increased with age for both males and females (Figure 2b).

Figure 3 shows ED-LOS was higher for females than males for both low-acuity (Figure 3a) and high-acuity (Figure 3b) patients. ED-LOS was significantly higher for those arriving by ambulance than on foot.

Figure 4 shows that while ED-LOS decreased with age for low-acuity patients, regardless of their mode of arrival (Figure 4a), the opposite was true for high-acuity patients (Figure 4b).

## Discussion

Since ED-LOS is one of the main performance indicators of hospital emergency services, measuring and understanding its main determinants is critical for managers from an operations perspective. This study analyzed the main and interaction effects of 4 determinants of ED-LOS (gender, age, mode of arrival, and clinical acuity). Previous studies have used various methodologies to study the main effects of different variables on ED-LOS: chi-square tests^6,7,8,11,16,18,19,25,26,27,28,30,32,37^ multiple linear regression,^10,12,13,20,22,23,24,26,36,39^ log linear regression,^21^ multilevel hierarchical analysis,^9,35,40^ autoregression models,^31^ simple/multivariate logistic regression models,^11,14,17,24,25,34,43^ ANOVA,^5,6,32^ and the Cox proportional hazard model.^29,33^ However, the literature lacks studies of the interaction effects of 2 or more factors. This study therefore used a between-subject factorial ANOVA to analyze both the main and interaction effects of at least 2 independent variables on the dependent variable for independent observations.

Many researchers have investigated the main effect of gender. Although some of concluded that it had a significant effect on ED-LOS,^5-7^ whereby female ED-LOS was significantly longer than male, many others have found no significant gender effect on ED-LOS.^8-14^ The present study supports the latter group. Although ED-LOS was longer for females than males, the difference was not significant.

The main effect of age has also been extensively analyzed, with most studies finding that age significantly affects ED-LOS,^5,9,11-13,15-21^ although some studies report an insignificant effect.^8,10,14,22^ The present study supports the majority of previous research in that age had a significant in that ED-LOS was longer for elderly patients.

In EDs, patients with high clinical acuity or more life-critical conditions are triaged into higher priority areas and vice versa for those with low acuity. As it should be, patients in higher priority areas are treated as soon as possible so their initial wait time to see a physician is shorter than that of other patient types. On the other hand, they generally require deeper investigations, diagnostic tests, or laboratory studies, which lengthens their ED treatment stay.^36,44^ Clinical acuity has therefore been extensively studied as an ED-LOS determinant, with a majority reporting that it is a significant ED-LOS determinant.^5,7-9,11,12,18,21,22,28-30^ Very few studies have reported a non-significant effect,^31^ with the findings in the present study support the majority of the literature in that clinical acuity was a significant determiner of ED-LOS.

Mode of arrival was another important and widely analyzed ED-LOS determinant. Mode of arrival can be classified as arriving by ambulance or on foot. Unsurprisingly, patients in life-critical situations generally arrive by ambulance. Thus, previous studies have found that mode of arrival significantly affects ED-LOS because patients arriving by ambulance require lots of investigations and longer stays.^11,12,17,19,21,22^ The present study supports these results.

The significant interaction between age and clinical acuity reported above shows that effect of age on ED-LOS differs for low and high acuity patients. Thus, while planning ED operations, such as generating estimations for ED-LOS, the patient’s age and clinical acuity should both be considered since an increase in age does not always a mean a longer ED-LOS. Figure 2a and 4a show that ED-LOS decreases with age for low acuity patients.

On the other hand, while the interaction in the present study between age and mode of arrival was significant when the model’s independent variables were gender, age, and mode of arrival, the interaction was not significant when the model included age, mode of arrival, and clinical acuity. This indicates that the interaction effects of the variables in the factorial ANOVA models are closely dependent on the set of independent variables considered in each model. The results in this study show that the effect of age on ED-LOS was different for patients arriving by ambulance and on foot (Figure 1) while age also had stronger and more significant effects for both high acuity and low acuity patients compared to those arriving by ambulance and on foot (Figure 4). Thus, when the model included the pairs of age, mode of arrival, and clinical acuity, the interaction between age and clinical acuity eliminated the interaction between age and mode of arrival.

One particularly interesting result concerns the age-related ED-LOS trend for low-acuity patients (Figure 2a). For these patients, ED-LOS was higher for young patients and lower for elderly patients, for both males and females. A possible interpretation of this result is that the past diagnoses of elderly patients are known (as most have a patient history in the hospital databases) whereas it takes time to determine the diagnosis in young patients (many of whom have no database record). In addition, the studied ED has a policy of prioritizing elderly patients whenever possible, even if they are low acuity. In contrast, Figure 2a also shows that older male patients, but not females, (age ≥65*)* have a longer ED-LOS than mid-age patients (age: [15-64]). Although this interesting result could not be specifically interpreted with the available data, it is probably related to differences in diagnoses given to each age group. Future studies could explore this result further by using patients’ diagnostic data.

## Limitations

This study had several limitations. First, the data was from just one institution so the findings may not be generalizable to other institutions. Although sample size was adequate for the research design, seasonal variations could not be included in the models because there was just one month’s data. Although the 4 factors considered here have been widely used in the literature to model ED-LOS, there are many other factors that previous studies have analyzed. Thus, using only 4 of these factors is another limitation of this study.

Despite the sample size, the limited number of considered factors, and the fact that it is a single site study, the results of this study indicate that other institutions should include interaction terms when analyzing factors that impact ED-LOS. As in this study, these other institutions may find significant interaction effects beyond the well-studied main effects that deserve further investigation while analyzing and modeling ED-LOS.

## Conclusion

Awareness of the important determinants of ED-LOS is critical for improving operations planning and service quality in emergency services. While decision-making can be improved by considering the main effects of each independent factor, it is also essential to focus on the interactions between different factors since these allow a deeper analysis for ED-LOS planning.

## Acknowledgments

The authors acknowledge hospital management for providing data to this study. The authors also acknowledge ED practitioners of this hospital for discussing their expertise and approving the findings of this study.

## Ethical issues

İzmir Katip Çalebi University, Non-interventional Clinical Studies, Institutional Review Board, approved in 19.04.2017 meeting.

## Competing interests

Authors declare that they have no competing interests.

## Authors’ contributions

GS: conception and design, analysis and interpretation of data, drafting manuscript, statistical analysis, supervision. MGA: acquisition of data, analysis and interpretation of data, critical revision of the manuscript for important intellectual content, administrative, technical, or material support. İK: critical revision of the manuscript for important intellectual content, administrative, technical, or material support.

## Authors’ affiliations

^1^Department of Business Administration, Yaşar University, İzmir, Turkey. ^2^Department of Emergency Medicine, Çiğli Regional Training Hospital, İzmir, Turkey. ^3^Department of General Surgery, Çiğli Regional Training Hospital, İzmir, Turkey.

## Supplementary Files


Supplementary file 1. Assumption check for between-subject factorial ANOVA

Supplementary file 2. Detailed Statistics on Hypothesis Testing.

## Key messages

**Implications for policy makers**

From an operations perspective, this study offers valuable insights for emergency department (ED) managers and practitioners:

- These findings may provide additional information for practitioners starting from the triage process while classifying patients based on their clinical acuity levels, relevant ED staff, and the patient and/or relatives can be informed about estimated length of stay (LOS).
- Estimated LOS values in each category can be used for planning, such as informing ambulance services regarding the number of high acuity patients requiring long stays, and efforts to persuade them to direct subsequent ambulances to another ED if possible.
- While planning the daily shifts of ED personnel, estimated LOS values can be used for increasing personnel numbers in higher priority areas when elderly patients arriving by ambulance enter the system, and increasing personnel numbers in lower priority areas when the number of young patients classified as low acute increases.
- For planning ED capacity in the medium to long term, these findings are useful for practitioners if combined appropriately with a demand forecasting model.

**Implications for the public**

One major problem during emergency department (ED) visits of patients is the overcrowded environment causing long stays and dissatisfaction. The findings of this study (using main and interaction effects of determinants of ED-length of stay (LOS), estimated LOS values for each category, and deciding which patient groups have the longest stays) will enable policy-makers to prepare better ED operational plans, such as demand forecasting, capacity planning, and scheduling. Improved performance in operational plans may decrease ED overcrowding, thereby providing patients with timely and high-quality medical care and thus increasing patient satisfaction.
