# Supplementary material for: Analyzing Main and Interaction Effects of Length of Stay Determinants in Emergency Departments
Source: Int J Health Policy Manag. 2019 Nov 16;9(5):198–205. doi: 10.15171/ijhpm.2019.107 (PMC7306116; doi:10.15171/ijhpm.2019.107)
Supplement: Supplementary file 1 — Assumption check for between-subject factorial ANOVA [file ijhpm-9-198-s001.pdf]

Supplementary file 1. Assumption Check for Between-Subject Factorial ANOVA

Assumption 1: The dependent variable should be measured at the continuous level (ED-LOS, the dependent variable of this study was measured at the continuous level).

Assumption 2: The independent variables should consist of 2 or more categorical independent groups (gender, mode of arrival, and the triage category all had 2 independent groups whereas age had 3 independent groups).

Assumption 3: Observations need to be independent, with no relationship between observations in each group (observations were independent; each entry of the data set represents a different patient).

Assumption 4: There should be no significant outliers (outliers were removed from the analysis in data pre-processing).

Assumption 5: The dependent variable should be approximately normally distributed for each combination of the groups of the independent variables: This assumption was violated. Kolmogorov Smirnov test results and histograms of ED-LOS for each levels of independent variables are presented below:

Table S1. Tests of Normality for ED-LOS

**Independent Variable**	**Levels**	**Kolmogorov Smirnov Test**			
		**Statistic**	**df**	***P* value**	**Result**
gender	female	0.149	14,837	p<0.001	ED-LOS is skewed
	male	0.162	14,376	p<0.001	ED-LOS is skewed
age	age ≤ 14	0.157	6,056	p<0.001	ED-LOS is skewed
	age: [15-64]	0.156	20,647	p<0.001	ED-LOS is skewed
	age≥65	0.072	2,510	p<0.001	ED-LOS is skewed
mode of arrival	walk in	0.156	27,884	p<0.001	ED-LOS is skewed
	by ambulance	0.042	1,329	p<0.001	ED-LOS is skewed
clinical acuity	high acuity	0.110	14,970	p<0.001	ED-LOS is skewed
	low acuity	0.167	14,243	p<0.001	ED-LOS is skewed

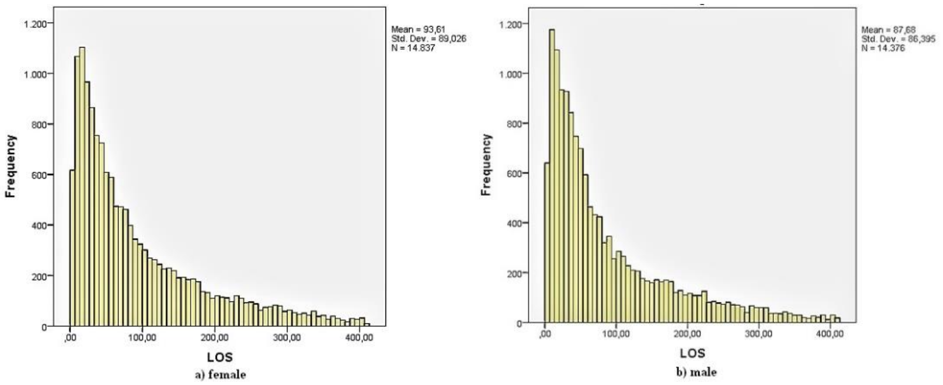

Figure S1. Histograms for Levels of Gender.

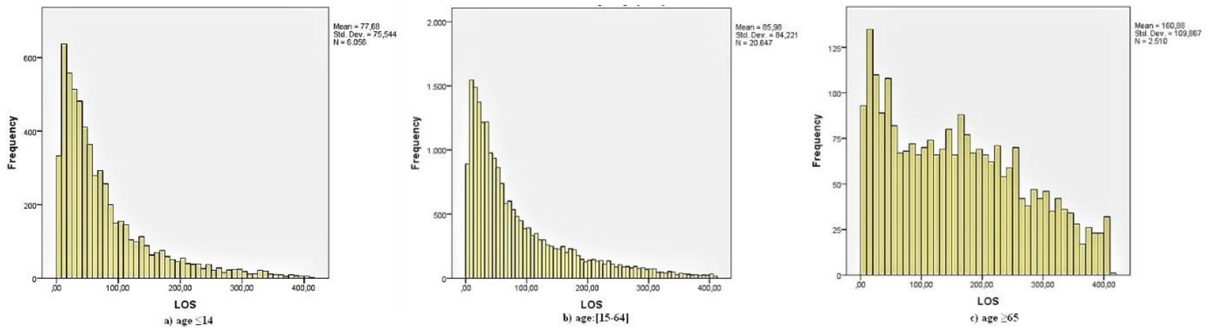

Figure S2. Histograms for Levels of Age.

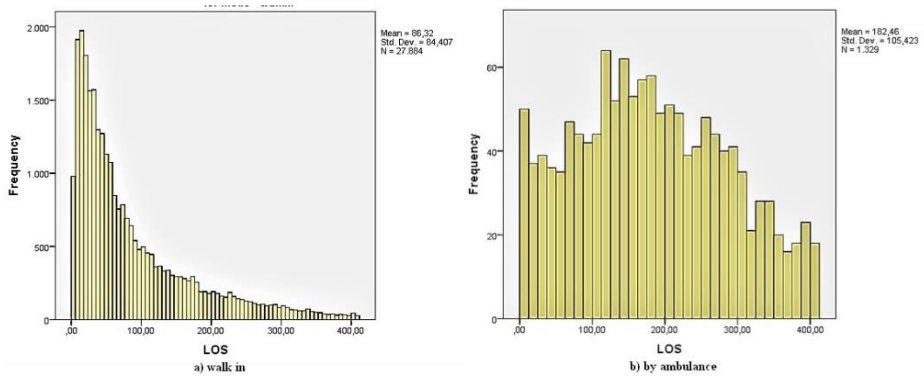

Figure S3. Histograms for Levels of Mode of Arrival.

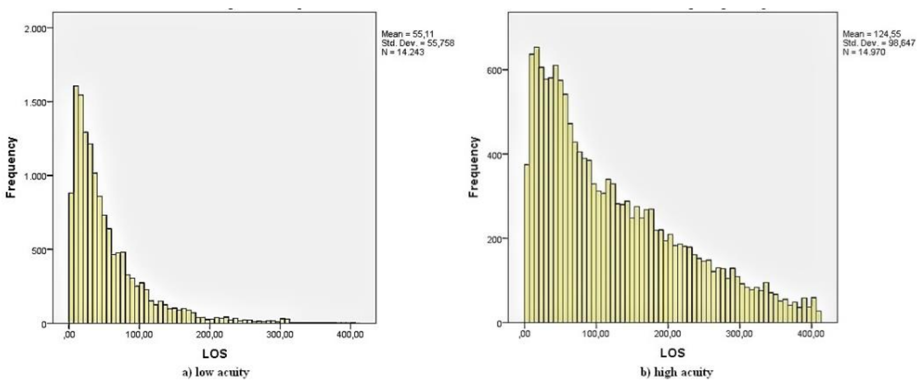

Figure S4. Histograms for Levels of Clinical Acuity.

Assumption 6: There needs to be homogeneity of variances for each combination of the groups of the independent variables: This assumption was violated. The results for Levene’s test for homogeneity of variances are presented below:

Table S2. Levene’s Test for Homogeneity of Variances

Set of hypotheses	Independent variables	F	df1	df2	*P* Value	Result
Set 1	gender, age, mode of arrival	87.315	11	29,201	p<0.001	Variances are not equal
Set 2	gender, age, clinical acuity	471.560	11	29,201	p<0.001	Variances are not equal
Set 3	gender, clinical acuity, mode of arrival	735.839	7	29,205	p<0.001	Variances are not equal
Set 4	age, clinical acuity, mode of arrival	492.106	10	29,201	p<0.001	Variances are not equal
