# Supplementary material for: Analyzing Main and Interaction Effects of Length of Stay Determinants in Emergency Departments
Source: Int J Health Policy Manag. 2019 Nov 16;9(5):198–205. doi: 10.15171/ijhpm.2019.107 (PMC7306116; doi:10.15171/ijhpm.2019.107)
Supplement: Supplementary file 2 — Detailed Statistics on Hypothesis Testing. [file ijhpm-9-198-s002.pdf]

Supplementary file 2. Detailed Statistics on Hypothesis Testing

independent variables of models	source	Type III sum of squares	Degrees of freedom	Mean square	F	Significance (p values)	R squared and adjusted R squared
gender, age, mode of arrival	corrected model	20,826,329.70	11	1,893,302.70	270.60	p<0.001	R squared= 0.093 Adjusted R squared=0.092
	intercept	31,389,773.19	1	31,389,773.19	4,486.34	p<0.001	
	gender	4,861.21	1	4,861.21	0.70	0.405	
	age	2,941,857.60	2	1,470,928.80	210.23	p<0.001	
	mode of arrival	1,890,506.05	1	1,890,506.05	270.20	p<0.001	
	gender and age	774.74	2	387.39	0.06	0.946	
	gender and mode of arrival	47.25	1	47.25	0.01	0.935	
	age and mode of arrival	258,492.73	2	129,246.37	18.47	p<0.001	
	gender, age and mode of arrival	11,535.15	2	5,767.57	0.82	0.439	
	error	204,311,864.88	29,201	6,996.74			
	total	465,438,755.03	29,213				
	corrected total	225,138,194.57	29,212				
gender, age, clinical acuity category	corrected model	41,766,117.31	11	3,796,919.76	604.64	p<0.001	R squared=0.186 Adjusted R squared= 0.185
	intercept	50,311,703.44	1	50,311,703.44	8,011.86	p<0.001	
	gender	15,484.07	1	15,484.07	2.47	0.116	
	age	646,849.07	2	323,424.53	51.50	p<0.001	
	clinical acuity	8,434,991.64	1	8,434,991.64	1,343.23	p<0.001	
	gender and age	34,822.95	2	17,411.47	2.77	0.063	
	gender and clinical acuity	447.25	1	447.25	0.07	0.790	
	age and clinical acuity	1,040,019.53	2	520,009.76	82.81	p<0.001	
	gender, age and clinical acuity	8,740.36	2	4,370.18	0.70	0.499	
	error	183,372,077.27	29,201	6,279.65			
	total	465,438,755.03	29,213				
	corrected total	225,138,194.57	29,212				
gender, mode of arrival, clinical acuity category	corrected model	40,549,418.5	7	5,792,774.08	916.51	p<0.001	R squared=0.180 Adjusted R squared= 0.180
	intercept	1,370,454.65	1	1,370,454.65	216.83	p<0.001	
	gender	1,100.90	1	1,100.90	0.174	0.676	
	clinical acuity	229,150.28	1	229,150.28	36.26	p<0.001	
	mode of arrival	4,9047.27	1	4,9047.27	7.76	0.005	
	gender and clinical acuity	494.81	1	494.81	0.08	0.780	
	gender and mode of arrival	8.03	1	8.03	0.00	0.972	
	clinical acuity and mode of arrival	17,035.78	1	17,035.78	2.70	0.101	
	gender, clinical acuity and mode of arrival	84.31	1	84.31	0.01	0.908	
	error	184,588,776	29,205	6,320.45			
	total	465,438,755	29,213				
	corrected total	225,138,194.6	29,212				
age , mode of arrival, clinical acuity category	corrected model	44,420,429.9	10	4,442,042.9	717.79	p<0.001	R squared=0.197 Adjusted R squared= 0.197
	intercept	1,397,096.23	1	1,397,096.23	225.76	p<0.001	
	age	507,720.220	2	253,860.11	41.02	p<0.001	
	clinical acuity	162,453.91	1	162,453.91	26.25	p<0.001	
	mode of arrival	33,770.56	1	33,770.56	5.46	0.019	
	age and clinical acuity	522,687.01	2	261,343.50	42.23	p<0.001	
	age and mode of arrival	663.77	2	331.89	0.05	0.948	
	clinical acuity and mode of arrival	2,561.20	1	2,561.20	0.41	0.520	
	age, clinical acuity and mode of arrival	3,288.71	1	3,288.71	0.53	0.466	
	error	180,717,764.7	29,202	6,188.54			
	total	465,438,755	29,213				
	corrected total	225,138,194.6	29,212				
